# Molecular characterization of mitochondrial COI gene sequences in *Micraspis allardi* from Pakistan

**DOI:** 10.1371/journal.pone.0294034

**Published:** 2023-12-27

**Authors:** Rida Asrar, Mariyam Masood, Imran Bodlah, Ghulam Rasool, Nazia Suleman, Sumaira Yousaf

**Affiliations:** 1 Institute of Physiology and Pharmacology, Faculty of Veterinary Science, University of Agriculture, Faisalabad, Pakistan; 2 Department of Zoology, Government College Women University, Faisalabad, Pakistan; 3 Insect Biodiversity and Conservation Group, Department of Entomology, Pir Mehr Ali Shah Arid Agriculture University, Rawalpindi, Pakistan; 4 Institute of Molecular Biology and Biotechnology (IMBB), The University of Lahore, Lahore, Pakistan; 5 Nuclear Institute for Agriculture and Biology, Faisalabad, Pakistan; Sakarya Uygulamali Bilimler Universitesi, TURKEY

## Abstract

The Coccinellidae is a highly diversified family of order Coleoptera. Coccinellid ladybirds are well known for their role as biological control agent against varied range of agricultural pests. The samples of coccinellid ladybird collected from Pakistan were identified and characterized as *Micraspis allardi* (Mulsant, 1866). This is one of the least-studied ladybird species with limited work on its ecological distribution as a biological control agent. The genus *Micraspis* has vast genetic diversity with a possible presence of unknown number of cryptic species. Sequence information of some species of the genus Micraspis are present in NCBI database. However, least molecular data or sequences describing *M*. *allardi* could be available from database. Therefore, morphological and molecular characterization was imperative for this species. Here, the samples collected from sugarcane field of Faisalabad District of Pakistan and were identified by using morphological and molecular protocols. For molecular identification, two different regions of mitochondrial cytochrome c oxidase I (COI) gene (COI-5′ and COI- 3′) were used as molecular markers for the identification of the species. Morphological appearance, DNA sequence similarity searches and phylogenetic analysis collectively indicated it as *M*. *allardi*. To the best of our knowledge, this is the first report providing molecular evidence of *M*. *allardi* using mitochondrial DNA barcode region (658bp) as well as mtCOI-3ʹ sequences (817bp). The study will help in understanding population genetics through diversity analysis, ecological role, and phenotypic structures associated with the geographic range of this species.

## Introduction

Ladybird beetles (Coleoptera; Coccinellidae) are found globally, with over 6,000 described species [1] classified in around 360 genera, of which 90% are regarded as beneficial with substantial agricultural significance in biological control programs [2, 3] *Micraspis allardi* (Mulsant, 1866) is a predatory coccinellid with ecological distribution in Indonesia, Pakistan India, Nepal, Myanmar, Bangladesh, and Bhutan [4–6] with a host range consisting mainly of plant hoppers [7], aphids [8, 9] and psyllids [10, 11]. Although ecology, diversity and distribution of this species has been reported from Pakistan [4, 12, 13]. Even though molecular sequences of some other species of *Micraspis* have also been reported and are present on NCBI data base. However, *M*. *allardi* has morphological descriptions based on phenotypic features only. So far, molecular data or sequences regarding characterization of *M*. *allardi* are not available on the NCBI website.

To better understand and utilize ladybird beetles as a bio-resource, proper classification and identification are required [14]. Different molecular markers, some particular DNA sequences, have been reported for the identification of species [15–17]. The mitochondrial cytochrome c oxidase subunit I (mtCOI) gene having variable sequences contributes a lot to species identification. However, some species showed limited sequence variability, while others display high level of polymorphisms, thus complicating species identification. A specific region from 5ʹ end of mtCOI gene (658 bp) has been recommended as a uniform DNA “barcode” region for the identification of species of all animals [18, 19]. Species recognition in *Coccinellini* is mainly based on morphological characters and DNA barcoding. The morphological identification of ladybird is difficult due to small size; however, DNA barcode identification is very effective and has been reported previously [20–22]. The identification of species using DNA barcode depends upon genetic distances and differences between intra as well as interspecific divergences [23, 24]. In addition, the aptness of the COI region to differentiate known species, identification of cryptic species as well as species-level phylogenetic relationships is imperative [19, 25]. Therefore, DNA barcoding using the 5ʹ region of mitochondrial COI DNA sequence is an effective and reliable tool for identifying specimens of unknown origin and taxonomic status [18, 26] belonging to multiple texa including *Coccinellidae* species [19, 27]. Bold Systems (Taxonomic browser) indicated very limited information for *M*. *allardi* with one specimen record from Pakistan but without molecular characterization. However, the NCBI data base showed partial sequences of COI-5ʹ region for *Coccinellidae sp*. (JF890394, JF890394, JF890535, JF890581) submitted from Pakistan.

Here, the collected specimens of beetle from Pakistan were identified as *M*. *allardi* according to morphological appearance as well as molecular characterization using mitochondrial cytochrome c oxidase subunit 1 (COI) gene sequences (mtCOI-3ʹ and mtCOI-5ʹ “barcodes region”). The availability of these gene sequences may allow the deposition of mitochondrial reference sequences of this species in commonly used repositories like the Barcode of Life database (BOLD), which is mainly used for molecular community analyses and help in species identification [28–30]. To use *M*. *allardi* as a biocontrol agent against sucking insect pests like aphids, plant hoppers, and whiteflies, insufficient information regarding *M*. *allardi* demands detailed sequence analysis and molecular characterization.

## Materials and methods

### Sample collection and morphological identification

Samples of beetle were collected from sugarcane crops at Faisalabad, Pakistan (Latitude: 31° 4’ 3.4104". Longitude: 72° 57’ 58.6872").

Morphological identification was performed with the help of main taxonomic characters provided in previous studies [31], based on morphological characteristics and male genitalia. The collected specimens were identified up to species level by comparison with other related identified species. Adult specimens and their genitalia were photographed with the help of digital camera (Amscope 18 megapixel camera) attached to LEICA MS5 microscope. The methodology for genitalia extraction and microphotography follows previous studies [31, 32] Some beetles were preserved in 80% ethanol for further processing of DNA extraction and molecular identification.

### DNA extraction and PCR amplification

Three independent samples were selected for molecular identification. Genomic DNA extraction was done using the CTAB method with some modifications and quantified the extracted DNA using nanodrop spectrophotometer (Thermo Scientific Multiskan GO^™^). PCR amplifications were carried out using universal primer pairs C1J2195/TL2N3014 (TTGATTTTTTGGTCATCCAGAAGT/ TCCAATGCACTAATCTGCCATATTA) and LCO1490/HCO2198 (GGTCAACAAATCATAAAGATATTGG /TAAAGTTCAGGGTGACCAAAAAATCA) for mtCOI-3ʹ and mtCOI-5ʹ regions, respectively [33, 34]. The PCR reaction mixture contained 2X DreamTaq Green PCR Master Mix (Thermo Fisher Scientific), primers, and beetle DNA. The PCR cycling parameters were one denaturation cycle of 94°C for 5 min, followed by 35 cycles of 94°C for 1 min, 40°C for 1 min, and 72°C for 1 min, followed by a final extension of 72°C for 7 min for the mtCOI-3ʹ fragment. While for the mtCOI-5ʹ fragment same profile was used but the annealing temperature was 45°C for 1 min.

### Cloning, sequencing and sequence analysis

The PCR products were ligated in the pTZ57R/T plasmid vector (Thermo Fisher Scientific), transformed into *E*.*coli* strain Top10 and confirmed by restriction analysis [26]. The confirmed clones were sequenced, and the sequences of each cloned insert were determined by bi-directional, automated Sanger dideoxy chain termination sequencing from Eurofins Genomics. The DNA sequences were assembled, aligned, and edited using Lasergene software (DNASTAR, Madison, WI). Sequence alignment and pairwise distance analyses were performed using MEGA7 [35]. The sequences were submitted to NCBI Genbank.

The model of evolution was selected based on a consensus of the Bayesian Information Criterion (BIC), the corrected Akaike Information Criterion (AICc), and the maximum likelihood parameters) using MEGA7 [35]. The General Time Reversal model (GTR+G+I), with gamma distributed rate of variation among sites being best-fit model of evolution for both mtCOI-3ʹ and mtCOI-5ʹ fragments was used. The phylogenetic trees for both mtCOI-3ʹ and mtCOI-5ʹ sequences were constructed with the sequences downloaded from the database separately using the Maximum Likelihood method [35]. 500 bootstrap replicates were used to test the reliability of the constructed tree. Phylogenetic trees were rooted with the *Bemisia tabaci* mtCOI-3ʹ and COI-5ʹ sequences as out-group.

## Results

### Morphological identification

#### Description

**Fig 1:**
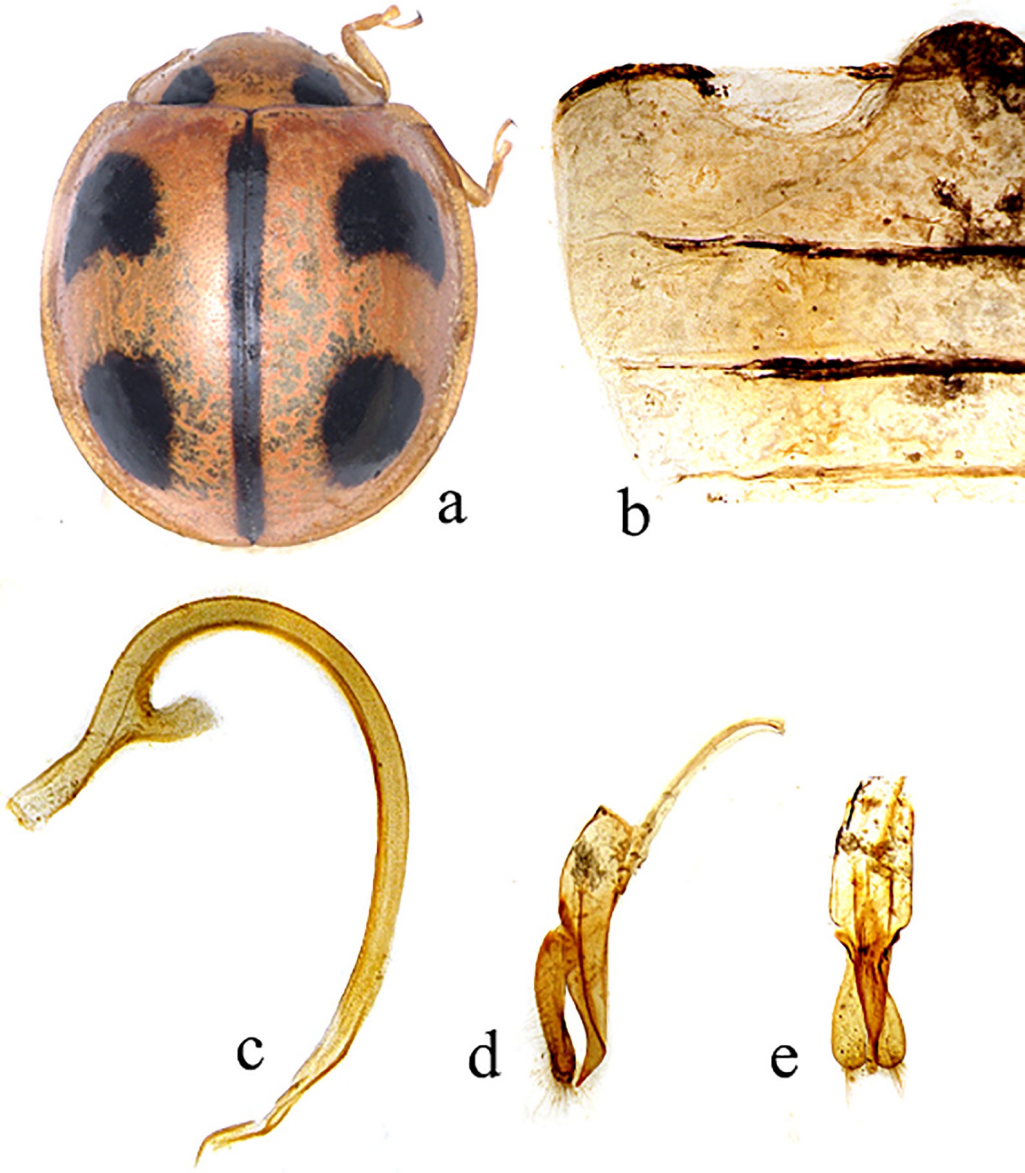
Morphological description of the *Micraspis allardi*. (a): Dorsal-view (b): Abdomen (c): Penis (d): Tegmen lateral-view (e): Tegmen ventral-view.

Body (Fig 1a) Dorsum glabrous, slightly round, moderately convex; head with mouth-parts and antennae brown; scutellum small and brownish; pronotum yellowish brown with two basal circular black spots; elytra brown and sometimes pink with black four spots; elytral suture with black strip; under surface brown; prosternal process without carinae; first abdominal ventrite (Fig 1b) with medially separate and incomplete post-coxal line.

#### Male genitalia

Penis (Fig 1c) is thin; the penis capsule (Fig 1d) has a long outer arm and short inner arm; the penis apex (Fig 1c) is blunt and has a small thread-like structure on the inner side. Tegmen (Fig 1d and 1e) are robust; the penis guide is broader at the base by about a third of its length, and the last two-thirds of its length gradually tapers into a slightly curved apex in lateral view (Fig 1d), but narrows abruptly into a blunt tip in ventral view (Fig 1e).

#### Comments

The following characteristics distinguish *Micraspis allardi* from other species in the genus *Micraspis*: The penis (Fig 1c) is narrow, and the penis guide (Fig 1d) is wider at its base than it is at its apex.

### Amplification of mitochondrial COI barcode and 3ʹ regions

The universal primers LCO1490/HCO2198 amplified and 658 bp product was obtained barcode 5ʹ region and primer pairs C1J2195/TL2N3014 produced 817 bp fragments from COI 3ʹ region. The cloned PCR products were confirmed using *EcoR*I and *Hind*III restriction enzymes revealed the same PCR sizes. Five more repetitive sequences of the confirmed clones were selected for the construction of a phylogenetic tree.

### Phylogenetic analysis of mtCOI-3ʹ and mtCOI-5ʹ sequences

**Fig 2:**
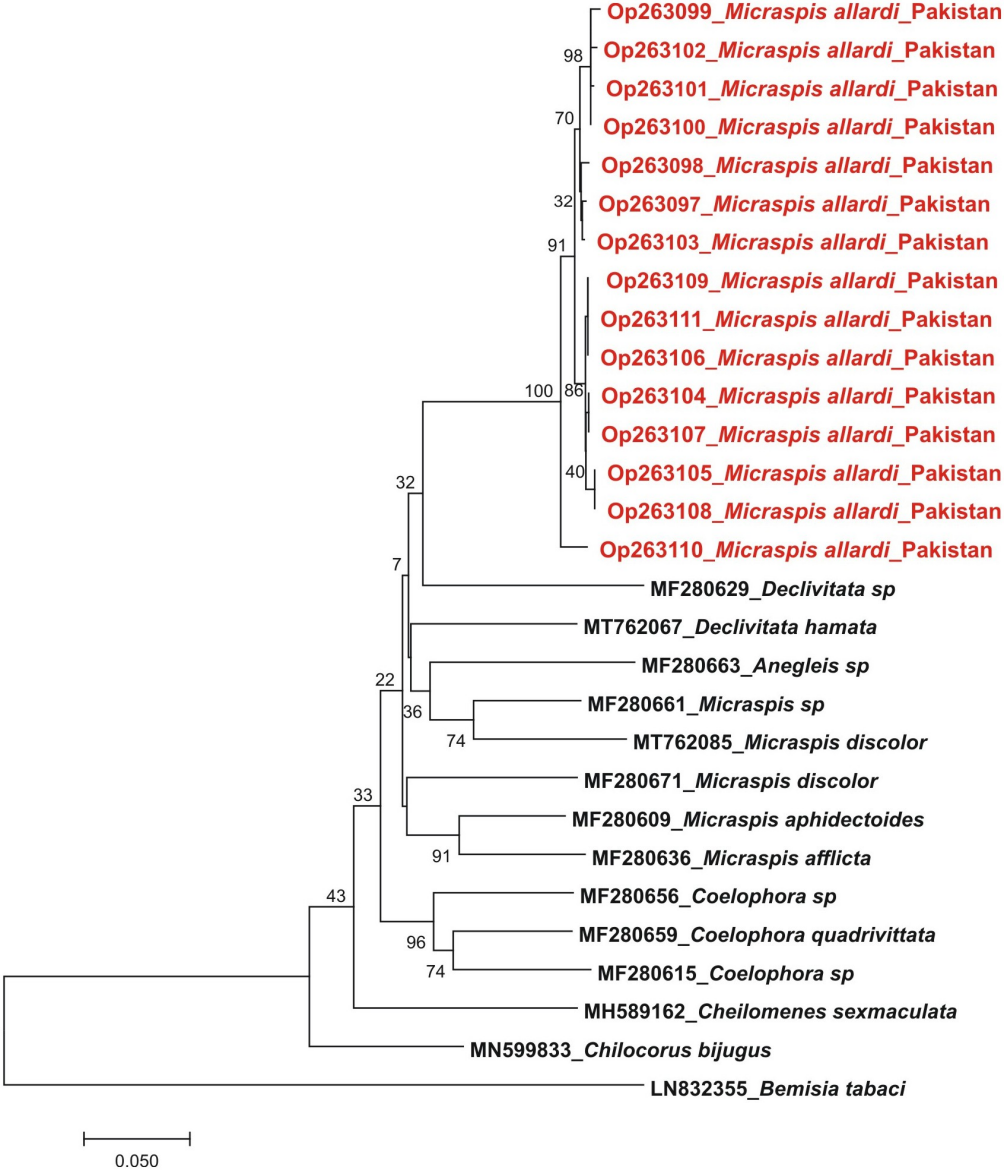
Phylogeny analysis of COI-3ʹ sequences. Phylogenetic tree analysis of under study beetle species (red colour) with sequences from the database.

The raw Sanger sequences were cleaned to remove restriction enzyme sites, trimmed to the same length, and aligned. We applied the Kimura-2-parameter (K2P) model for DNA barcoding analysis. This study reports five sequences of mtCOI-5ʹ with accession numbers OP263122- OP263126 and fifteen sequences of mtCOI-3ʹ region, submitted to database with accession numbers OP263097- OP263111. To find out the sequence similarity of mtCOI-5ʹ and mtCOI-3ʹ sequences, BLAST search was used. The most similar sequences were downloaded from the databases. During the BLAST search of mtCOI-3ʹ sequences, no reference sequence having a similarity of more than 85–87% was found in the database (Fig 2). Therefore, this is the first report of the mtCOI-3ʹ sequence of the beetle sample identified as *Micraspis allardi*.

**Fig 3:**
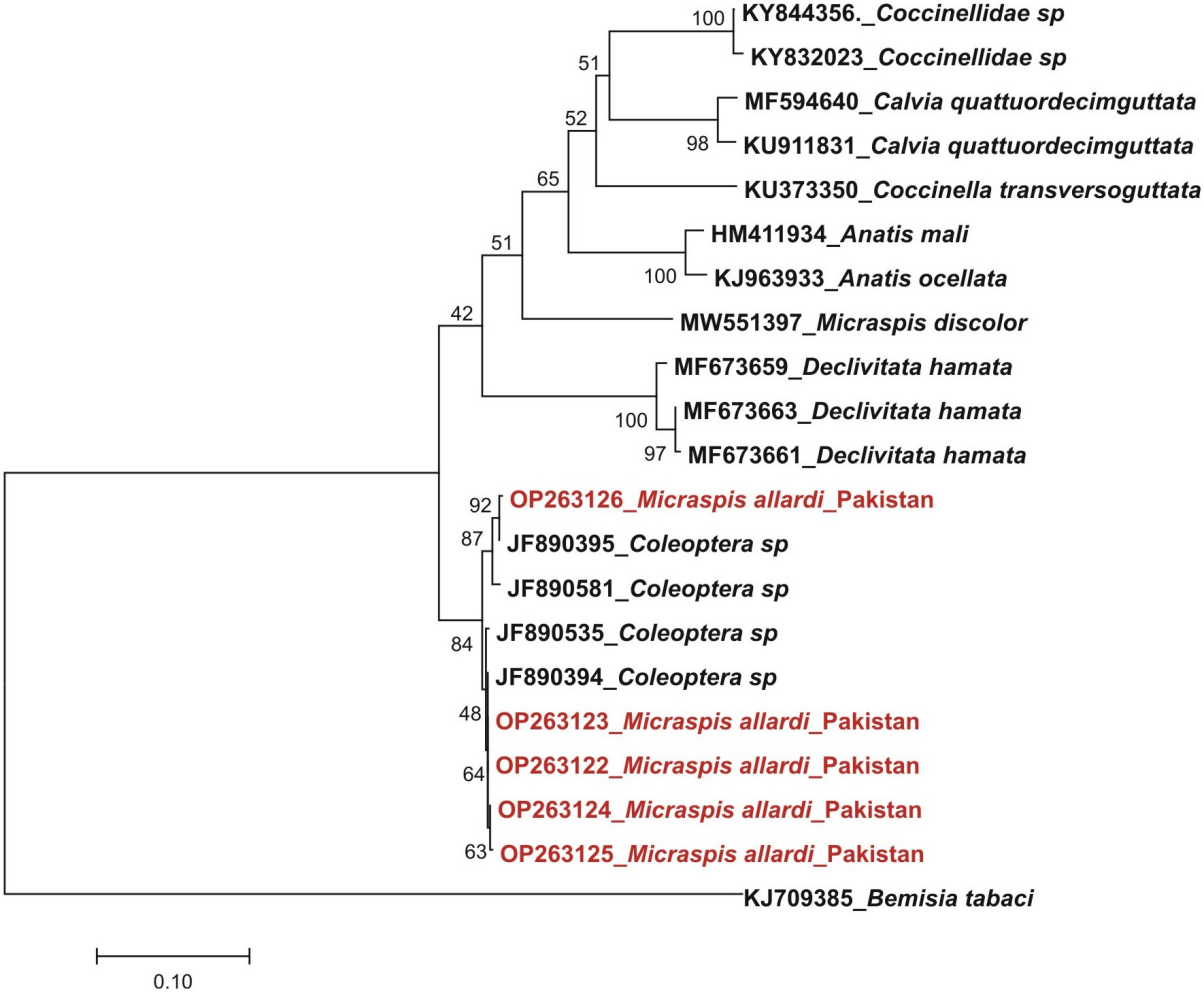
Phylogenetic analysis of COI-5ʹ sequences. Phylogenetic tree analysis of under study beetle species (red colour) with sequences from the database.

However, BLAST search of mtCOI-5ʹ (barcode) sequences showed 100% similarity with the four sequences in database having Accession Nos. JF890394, JF890395, JF890535 and JF890581 but named as Coccinellidae sp. BOLD: AAP7928 (unpublished data). The analysis of our sequences alogwith the morphological identification, phylogenetic analysis and, and the sequences of mtCOI-5 collected from BOLD systems determined that beetle species described here is *M*. *allardi*. The sequences considerably expand the known morphological disparity of the species. Phylogenetic analysis of our samples also confirmed that JF890394, JF890395, JF890535, and JF890581 are the sequences of *M*. *allardi* (Fig 3). Sequence analysis of both fragments confirmed that the under-study beetle species belongs to the *M*. *allardi*. This study firstly reported the molecular identification and characterization of *M*. *allardi* using two fragments (mtCOI-3 and mtCOI-5) of mitochondrial COI gene sequences.

### Distance analysis

Regarding COI-3ʹ sequences obtained for our samples, the intraspecific distance ranged from 0.0–3.5% while the mean distance was 1.5%. A maximum interspecific distance was found to be 3.5% for the accession OP263110. However, for the COI-5ʹ sequences of the study, the intraspecific distance ranged from 0.0–2.1% while the mean distance was 0.8%. Thus, the distances among sequences showed that the sequences under study are of same species, *M*. *allardi*.

## Discussion

Here we present the first mitochondrial cytochrome c oxidase subunit 1 (COI) gene sequences COI-3ʹ and COI barcodes of *M*. *allardi*. Previous molecular work on *Micraspis* species has provided information regarding other species. The genus *Micraspis*) comprises vast genetic diversity, yet the unknown number of cryptic species may be present and should be reviewed using morphological and molecular characterization. The family Coccinellidae comprises two subfamilies: Microweiseinae Leng, and Coccinellinae Latreille also Coccinellinae consists of two tribes: Coccinellini and Chilocorini [36]. Coccinellidae contains a group of beetles that are considered important for their role in biological control [37; 38] and their diversity in varied habitats [5]. Recently, *M*. *allardi* (Coleoptera: Coccinellidae) has been reported as a biological control agent of *P*. *Perpusilla*, a sugarcane crop pest [39]. We also collected samples from sugarcane crops during a biological control study from the vicinity of Faisalabad. Previously its presence has been reported from the Faisalabad region [9, 40]. The collected sample of *M*. *allardi* has been identified using the morphological characteristics in Pakistan as described earlier [41] however other species have been characterized at molecular level [42–44].

So far, the species recognition of *M*. *allardi* is by morphological characteristics only as no data regarding molecular identification is available. While investigating the BOLD system [30], there is only one record from Pakistan (http://v3.boldsystems.org/index.php/Taxbrowser_Taxonpage?taxid=393866). However, there are four sequences of barcode region submitted in NCBI database (http://v3.boldsystems.org/index.php/Taxbrowser Taxonpage?taxid = 105896). The mtCOI-5ʹ resultant sequences of this study had sequence similarity identical to partial sequences of COI-5ʹ region for Coccinellidae sp (JF890394, JF890395, JF890535, JF890581). To date, no mtCOI-3ʹ sequences of *M*. *allardi* have been submitted and published. This first report uses the nucleotide sequences of two mitochondrial cytochrome oxidase I gene regions, COI-3ʹ and COI-5ʹ also termed as barcode region. Characterization of *M*. *allardi* using this technique provided accurate identification of the subject specie for future investigations and various research areas. Similar to previous reports, this study also utilized the COI gene for species identification, considered the best tool for identifying economically important species. Our study adds the first mitochondrial COI sequences of *M*. *allardi* to databases and thereby makes mitochondrial barcoding available for further studies on this important biocontrol agent. Future studies should build reference databases based upon *M*. *allardi* COI and other molecular marker genes combined with the morphological identification of species from diverse locations.

## Conclusion

This study provides the first sequences of *M*. *allardi* based upon mitochondrial COI marker. These sequences will be helpful for DNA barcoding of this biocontrol agent worldwide.
